# Insurance status impacts survival of hepatocellular carcinoma patients after liver resection

**DOI:** 10.1002/cam4.6339

**Published:** 2023-07-16

**Authors:** Zhancheng Qiu, Weili Qi, Youwei Wu, Lingling Li, Chuan Li

**Affiliations:** ^1^ Division of Liver Surgery, Department of General Surgery West China Hospital, Sichuan University Chengdu China; ^2^ Department of Information Management Center West China Hospital, Sichuan University Chengdu China

**Keywords:** hepatectomy, hepatocellular carcinoma, insurance, survival

## Abstract

**Background:**

This study intends to examine the effect of public insurance status on survival outcomes of HCC patients after liver resection in China.

**Methods:**

We divided 2911 HCC patients after liver resection included in our study into the Urban Employed‐based Medical Insurance group (UEBMI group, *n* = 1462) and the non‐Urban Employed‐based Medical Insurance group (non‐UEBMI group, *n* = 1449). A propensity score matching (PSM) analysis was used to control confounding factors. Overall survival (OS) was estimated by Kaplan–Meier curves and Cox proportional hazard models based on variables screened by Lasso regression. Competing risk analysis was used to analyze cancer‐specific survival (CSS).

**Results:**

UEBMI group had more male patients (*p* = 0.031), patients in the UEBMI group were older (*p* < 0.001) and had lower Charlson Comorbidity Index scores (CCI score, *p* < 0.001). Meanwhile, patients in the UEBMI group had better liver function (albumin‐bilirubin grade I [ALBI I], *p* < 0.001) and lower tumor burden (α‐fetoprotein [AFP], *p* = 0.009; Barcelona Clinic Liver Cancer stage [BCLC], *p* = 0.026; Milan criteria, *p* < 0.001; tumor size, *p* < 0.001; microvascular invasion [MVI], *p* = 0.030; portal vein tumor thrombosis [PVTT], *p* = 0.002). More patients in the UEBMI group received laparoscopic surgery (*p* = 0.024) and adjuvant transarterial chemoembolization (TACE, *p* < 0.001). After PSM, patients in the two matched groups had similar characteristics. Patients with recurrent HCC in the UEBMI were more likely to receive curative therapy (*p* < 0.001) and less likely to receive supportive care (*p* < 0.001). HCC patients after liver resection in the non‐UEBMI group had a worse OS before (*p* < 0.0001) and after PSM (*p* = 0.002). [Correction added on August 16, 2023 after first online publication. The p value has been updated in the preceding sentence.] In our Lasso‐Cox risk regression model, public health insurance status was an independent factor linked with OS (non‐UEBMI vs. UEBMI, hazard ratio [HR]: 1.27; 95% confidence interval [CI]: 1.12–1.46; *p* < 0.001). In the competing risk analysis, patients in the UEBMI group had a lower cumulative incidence of CSS before (*p* < 0.001) and after PSM (*p* = 0.001), and public insurance status of HCC patients after liver resection remained independently associated with CSS (non‐UEBMI vs. UEBMI; HR:1.36; 95% CI: 1.18–1.58; *p* < 0.001).

**Conclusions:**

Underinsured HCC patients after liver resection had worse survival outcomes. Less access to care for underinsured patients may explain the difference in survival, but the corresponding conclusions need to be further explored.

## 1 INTRODUCTION

Hepatocellular carcinoma (HCC) places a heavy burden on health systems worldwide.^1^ Although the treatment strategies for HCC patients are diverse based on tumor burden and liver function, surgical resection is a recognized and important means of achieving long‐term survival for HCC patients.^2^, ^3^ However, it is worth noting that HCC patients after surgery face an extremely high risk of recurrence (40%–70% in 5 years), meaning that a large proportion of patients will face treatments after recurrence, which can be a heavy financial burden for those who have already had one surgery.^2^, ^4^ Studies have reported that when patients face financial burdens, they may change their treatment regimen on their own to reduce medical costs, which may result in a poor prognosis.^5^

The provision of health insurance is vital to achieving universal health coverage and health equity in a nation because it can significantly reduce the financial burden on patients and positively impact the diagnosis and prognosis of diseases.^6^, ^7^, ^8^, ^9^, ^10^, ^11^, ^12^ A previous study reported that locally advanced gastric cancer patients undergoing gastrectomy who have limited or no insurance have a lower overall survival rate.^13^ Similar results were reported in esophageal cancer and lung cancer.^14^, ^15^ However, it is still unknown whether public health insurance status affects the prognosis of HCC patients after liver resection.

Public health insurance in China was initially divided into three types: Urban Employed‐based Medical Insurance (UEBMI), Urban Resident‐based Medical Insurance (URBMI) and New Rural Cooperative Medical Schemes (NRCMS). From 2009, URBMI and NRCMS have been amalgamated into Urban and Rural Resident Basic Medical Insurance (URRBMI) to reduce the gap between urban and rural health care.^16^ However, there are still disparities in the benefit packages of different forms of public health insurance. It is unclear whether public health insurance status affects the survival outcomes of HCC patients after liver resection. Thus, this research aims to examine the effect of public insurance status on survival outcomes of HCC patients after liver resection based on Chinese demographic features.

## 2 MATERIALS AND METHODS

### 2.1 Patients

**FIGURE 1:**
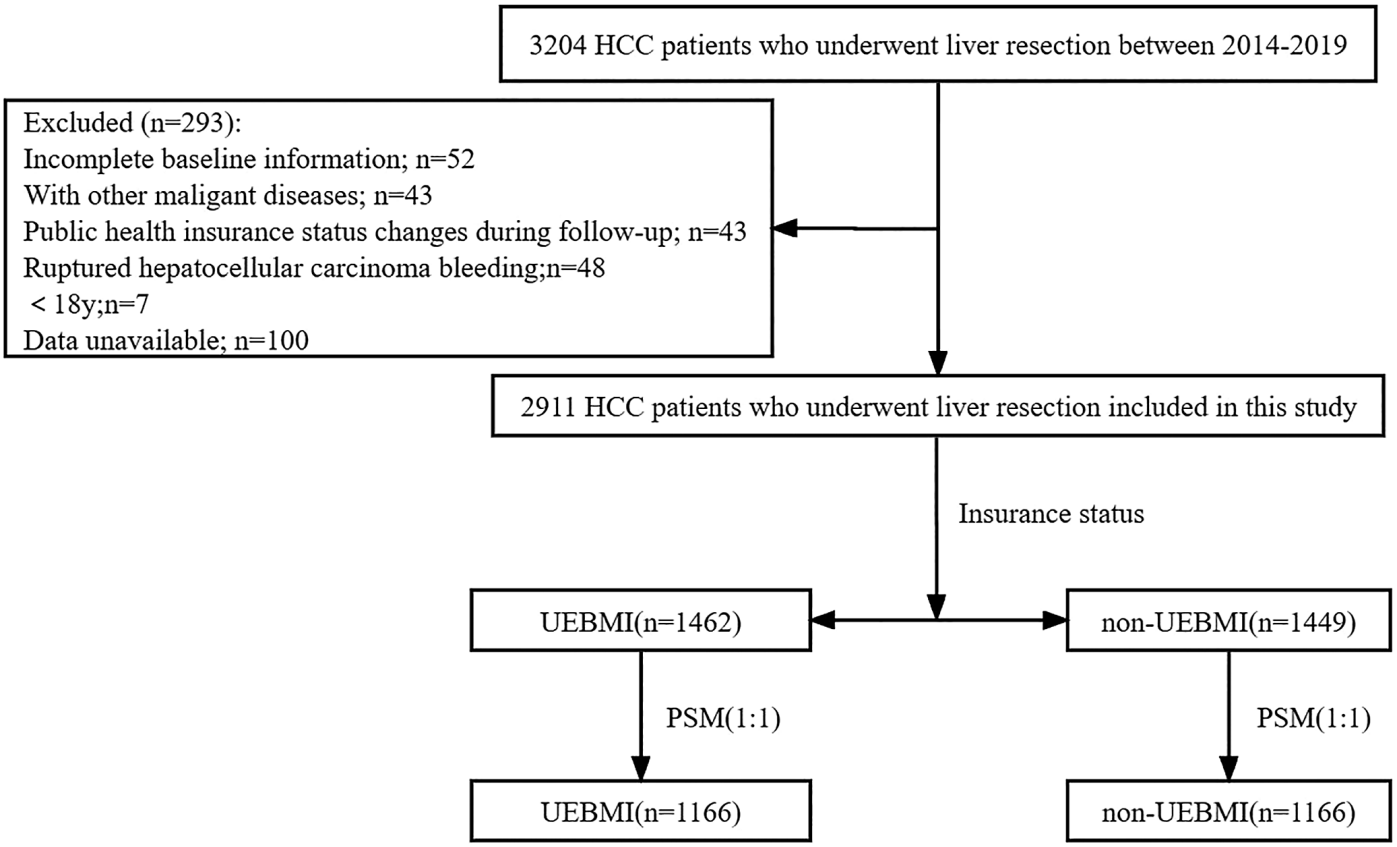
Sample selection flowchart. [Correction added on August 16, 2023 after first online publication. Figure 1 has been updated in this version.]

Three thousand two hundred four HCC patients who underwent liver resection at West China Hospital (WCH), Sichuan University, from 2014 to 2019 were enrolled in our study. All baseline information of the patients in our study was retrospectively collected from WCH's Hospital Information System (HIS) and Information Management System (IMC). The exclusion criteria were as follows: (1) incomplete baseline information; (2) ruptured hepatocellular carcinoma bleeding; (3) combined with other malignancies; (4) less than 18 years old; and (5) public health insurance status changes during follow‐up. Finally, our study included 2911 HCC patients who underwent hepatectomy (Figure 1). All patients included in the analysis were followed up after liver resection until death or May 31, 2022, using the passive follow‐up method (telephone contact and outpatient visit information). Our study focused on overall survival (OS) and cancer‐specific survival (CSS). OS was defined as the time from surgery to death from any cause or the last follow‐up. CSS was defined as the time from surgery to cancer‐specific death and censoring at the last follow‐up or non‐cancer‐specific death.

### 2.2 Health insurance

The UEBMI was launched for urban employment in 1998, and in an effort to extend health insurance coverage, the NRCMS for rural populations and the URBMI for urban inhabitants were established in 2003 and 2007, respectively.^17^ Payroll taxes of employes' wages are the primary funding resource for UEBMI, while the funding resource of URBMI and NRCMS mainly comes from government subsidies. Meanwhile, the three public medical insurance programs are administered and operated separately at the national and local levels. NRCMS is managed by China's National Health and Family Planning Commission (formerly China's Ministry of Health), and UEBMI and URBMI are managed by China's Ministry of Human Resources and Social Security.^18^ Thus, the benefit packages, which include drug provision, medical services, and reimbursement, are not equal across different public health insurance types.^19^ In general, with regard to benefits, UEBMI outperforms other types of medical insurance, such as URBMI and NRCMS.^7^ To assure the equity of urban and rural medical care, URBMI, and NRCMS have been merged into URRBMI, but disparities in benefits remain. Therefore, in our study, HCC patients after liver resection were separated into two groups based on the disparities in the benefits of public health insurance: the UEBMI group (*n* = 1462) and the non‐UEBMI group (*n* = 1449) (including URBMI: *n* = 380; NRCMS: *n* = 804; URRBMI: *n* = 265).

### 2.3 Statistical analyses

Continuous variables were described as the means ± SDs or median and quartile. Two‐sample *t* tests or Mann–Whitney *U* tests were used for comparison of continuous variables. Numbers and percentages were used to describe the categorical variables, and chi‐squared tests or Fisher's exact tests were used for comparison of categorical variables. A 1:1 propensity score matching (PSM) analysis was employed to control confounding factors. A caliper of 0.02 was used to generate matched patients based on propensity scores. The multivariate Cox regression model was built using variables screened by the least absolute shrinkage and selection operator (Lasso) regression to identify factors that were independently associated with OS. In the Lasso regression, an L1 penalty was applied to reduce some regression coefficients to exactly zero, and 10‐fold cross‐validation with minimal criteria was used to determine the optimal log (λ). The Kaplan–Meier method was used to compare the difference in OS of HCC patients after liver resection between the two groups. Comparing the variations in patients' CSS between the two groups was done using competing risk analysis. In order to compare the CSS differences between the non‐UEBMI group and the UEBMI group, the Gray approach was applied. The Fine and Gray model's multivariable competing risk analysis was utilized to identify independent CSS‐related components. Version 4.2.1 of the R statistical software was used for statistical analyses. A *p* value <0.05 was statistically significant.

## 3 RESULTS

### 3.1 Patient characteristics

Two thousand nine hundred and eleven (*n* = 2911) HCC patients were included in our research, with the UEBMI group comprised of 1462 (50.2%) patients and the non‐UEBMI group comprised of 1449 (49.8%) patients. Table 1 presents the characteristics of the patients in the two groups before PSM. HCC patients had a mean age of 53.0 years in the entire group (UEBMI vs. non‐UEBMI: 54.4 vs. 51.6, *p* < 0.001), and 2465 (84.7%) patients were male (UEBMI vs. non‐UEBMI: 86.1% vs. 83.2%, *p* = 0.031). HCC patients in the non‐UEBMI group had a higher CCI score (*p* < 0.001). Meanwhile, patients in the UEBMI group were diagnosed more recently (*p* = 0.013) and had lower levels of ALBI grade (*p* < 0.001), lower levels of AFP (*p* = 0.009), smaller tumor size (*p* < 0.001), earlier tumor stage (*p* = 0.026), and lower rates of MVI (*p* = 0.030) and PVTT (*p* = 0.002). Patients in the UEBMI group were more likely to meet the Milan criteria (*p* < 0.001) and more patients in the UEBMI group underwent laparoscopic surgery (*p* = 0.024). However, patients in the non‐UEBMI group were more likely to undergo major hepatectomy (*p* < 0.001) and less likely to receive adjuvant TACE therapy (*p* < 0.001). To control confounding factors, age, sex, smoking status, alcohol consumption, CCI score, year of diagnosis, ALBI grade, AFP, tumor size, type of surgery, major hepatectomy, adjuvant TACE, BCLC stage, Milan criteria, MVI, capsular invasion, cirrhosis, and PVTT were used as covariables for PSM. Table 1 also shows the characteristics of patients between the two matched group, and none of the variables between the two groups remained statistically distinct.

**TABLE 1:** Baseline characteristics for HCC patients who underwent liver resection.

	Entire cohort (*n* = 2911)			PSM cohort (*n* = 2332)		
Variables	UEBMI (*n* = 1462)	Non‐UEBMI (*n* = 1449)	*p* value	UEBMI (*n* = 1166)	Non‐UEBMI (*n* = 1166)	*p* value
Age, years	54.4 ± 12.1	51.6 ± 10.4	<0.001	52.8 ± 12.0	52.7 ± 10.0	0.675
Male	1259 (86.1%)	1206 (83.2%)	0.031	991 (85.0%)	988 (84.7%)	0.862
Smoke	749 (51.2%)	793 (54.7%)	0.059	634 (54.4%)	628 (53.9%)	0.803
Alcohol	354 (24.2%)	373 (25.7%)	0.341	292 (25.0%)	296 (25.4%)	0.849
CCI score			<0.001			0.670
1	929 (63.6%)	949 (65.5%)		749 (64.2%)	759 (65.1%)	
2	144 (9.8%)	81 (5.6%)		88 (7.5%)	77 (6.6%)	
≥3	389 (26.6%)	419 (28.9%)		329 (28.2%)	330 (28.3%)	
Year of diagnosis			0.013			0.984
2014–2015	426 (29.1%)	495 (34.1%)		360 (30.9%)	357 (30.6%)	
2016–2017	565 (38.7%)	530 (36.6%)		442 (37.9%)	446 (38.3%)	
2018–2019	471 (32.2%)	424 (29.3%)		364 (31.2%)	363 (31.1%)	
Married	1383 (94.6%)	1371 (94.6%)	0.980	1095 (93.9%)	1104 (94.7%)	0.422
HBV‐related	1396 (95.5%)	1396 (96.3%)	0.243	1119 (96.0%)	1125 (96.5%)	0.514
Child‐Pugh A/B	1455/7	1433/16	0.057	1159/7	1158/8	0.796
ALBI^a^ I/II	1192/270	1095/355	<0.001	916/250	936/230	0.306
AFP, >400 ng/L	1328 (90.8%)	1394 (93.4%)	0.009	1083 (92.9%)	1077 (92.4%)	0.635
Tumor size, cm	4.7 (3.1,7.1)	5.4 (3.3,8.8)	<0.001	4.8 (3.1,7.5)	5.0 (3.1,8.0)	0.337
Single tumor	1325 (90.6%)	1294 (89.3%)	0.234	1052 (90.2%)	1044 (89.5%)	0.583
BCLC stage						
0/A/B/C	110/1124/97/131	92/1078/103/176	0.026	92/872/84/118	84/884/82/116	0.922
Milan criteria^b^	740 (50.6%)	616 (42.5%)	<0.001	563 (48.3%)	551 (47.3%)	0.619
Laparoscopic/open hepatectomy	260/1202	213/1236	0.024	195 (16.7%)	187 (16.0%)	0.654
Major hepatectomy^c^	550 (37.6%)	669 (46.2%)	<0.001	472 (40.5%)	485 (41.6%)	0.584
Length of stay	10 (8,13)	10 (8,13)	0.443	10 (8,13)	10 (8,12)	0.504
Adjuvant TACE	300 (20.5%)	222 (15.3%)	<0.001	204 (17.5%)	204 (17.5)	1.000
Low differentiation	178 (12.2%)	209 (14.4%)	0.074	145 (12.4%)	148 (12.7%)	0.851
Capsular invasion	789 (54.0%)	787 (54.3%)	0.851	607 (52.1%)	633 (54.3%)	0.281
MVI	418 (28.6%)	468 (32.3%)	0.030	340 (29.2%)	351 (30.1%)	0.885
Satellite nodules	148 (10.1%)	173 (11.9%)	0.118	123 (10.5%)	132 (11.3%)	0.550
Cirrhosis	610 (41.7%)	643 (44.4%)	0.149	504 (43.2%)	515 (44.2%)	0.646
PVTT	104 (7.1%)	149 (10.3%)	0.002	95 (8.1%)	97 (8.3%)	0.880

Abbreviations: AFP, alpha‐fetoprotein; BCLC, Barcelona Clinic Liver Cancer staging system; CCI, Charlson Comorbidity Index; HBV‐related, hepatitis B‐related hepatocellular carcinoma; MVI, microvascular invasion; PVTT, portal vein tumor thrombosis; TACE, transarterial chemoembolization.

^a^ ALBI grades were classified into three levels (grades I, II, III = ≤−2.60, <−2.60 to ≤−1.39, >−1.39) based on the ALBI score: ALBI score = [(log10 bilirubin (in μmol/L) × 0.66) + (albumin (in g/L) × −0.085)].

^b^ Milan criteria was defined as up to three HCC nodules, the largest <3 cm in diameter or a single HCC nodule up to 5 cm in diameter.

^c^ Major hepatectomy was defined as was defined as the resection of more than three contiguous Couinaud segments.

In the overall cohort, 1488 patients suffered recurrence, with 754 (51.6%) patients in the UEBMI group and 734 (50.7%) patients in the non‐UEBMI group developing recurrence. Table 4 presents the difference in treatment for recurrence of patients with recurrent HCC in the UEBMI group and non‐UEBMI group. Significantly more recurrent HCC patients in the UEBMI group were treated with curative treatment (*p* < 0.001) and systemic therapy (*p* = 0.030). Recurrent HCC patients with non‐UEBMI were more likely to select supportive care (*p* < 0.001).

### 3.2 Influence of public health insurance status on overall survival and cancer‐specific survival

**FIGURE 2:**
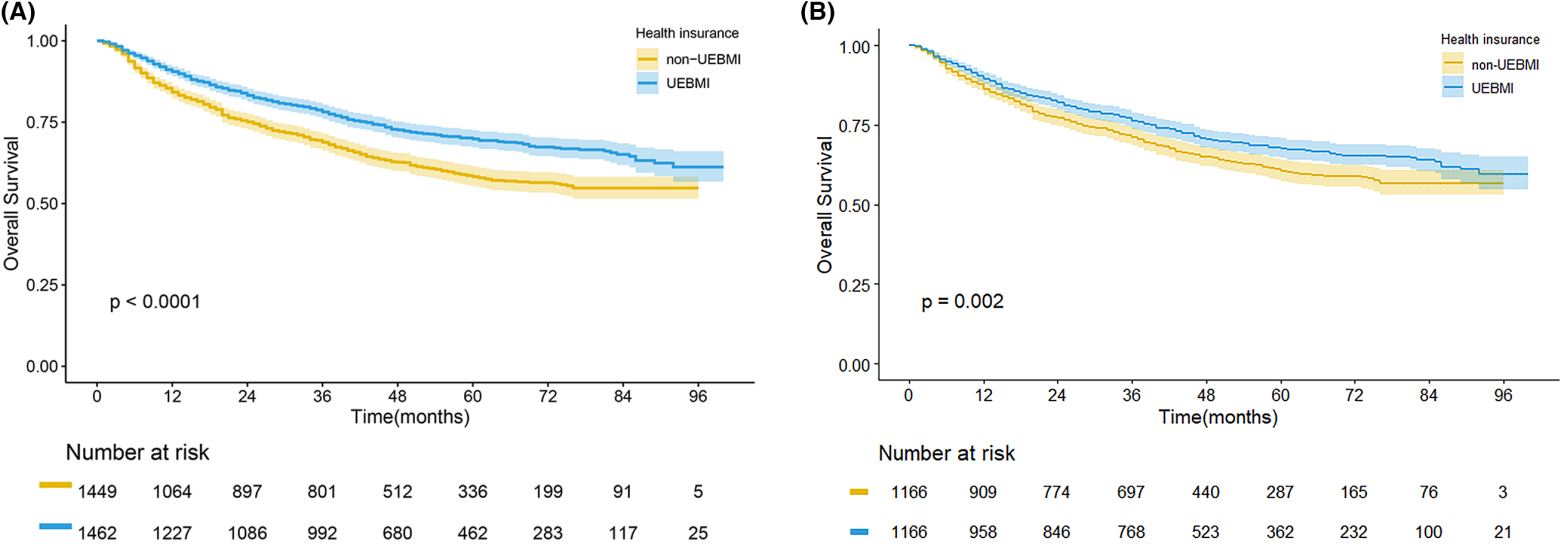
Kaplan–Meier curve of HCC patients after surgery in the UEBMI group and non‐UEBMI group; (A) Overall survival before PSM; (B) Overall survival after PSM. [Correction added on August 16, 2023 after first online publication. Figure 2B has been updated in this version.]

The median follow‐up time for patients was 55 months. The 5‐year OS rate was 64.3% in the entire cohort. In the UEBMI and non‐UEBMI groups, the 5‐year OS rates were 69.4% and 58.3%, respectively. After Kaplan–Meier analysis in our study, we found that HCC patients after liver resection in the UEBMI group had a better OS than those in the non‐UEBMI group (*p* < 0.0001, Figure 2A). In the two matched groups, OS disparities of HCC patients after liver resection still existed (*p* = 0.002; Figure 2B). [Correction added on August 16, 2023 after first online publication. The p value has been updated in the preceding sentence.]

**FIGURE 3:**
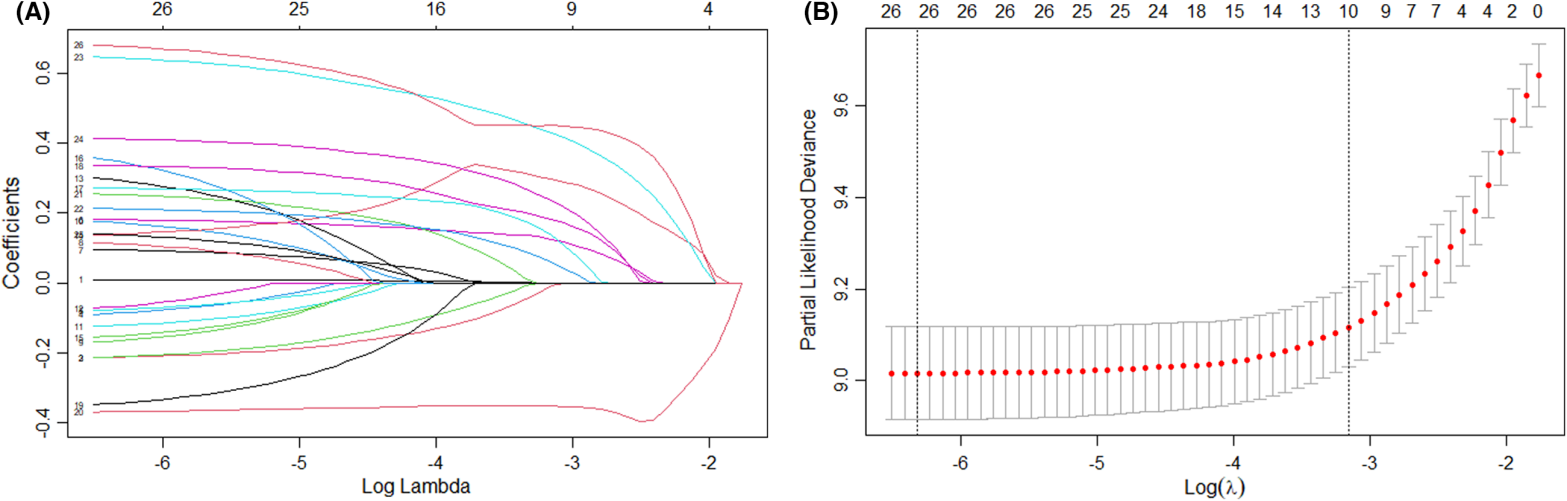
Screening of variables based on Lasso regression. (A) The variation characteristics of the coefficient of the variables of OS. (B) Identification of the optimal penalization coefficient λ in the Lasso model of OS. *Screening of the variables included in Table 1, and the variables screened by the Lasso regression model including insurance type, ALBI grade, tumor size, tumor stage, Milan criteria, major hepatectomy, capsule invasion, MVI, satellite nodules, and PVTT were incorporated in the Cox model.

The Lasso‐Cox proportional risk regression model including 2911 patients was performed to determine independent factors associated with OS of HCC patients after liver resection (Table 2; Figure 3A,B). Figure 3A shows the coefficient variation of the Lasso regression‐screened variables. The 10‐fold cross‐validation approach was used for the iterative analysis, and a model with excellent performance and a minimal number of variables was produced when λ was 0.039 (log (*λ*) = −1.409) (Figure 3B). The screened variables included insurance type, ALBI grade, tumor size, tumor stage, Milan criteria, major hepatectomy, capsule invasion, MVI, satellite nodules, and PVTT. In our Lasso‐Cox risk regression model, HCC patients after liver resection covered by non‐UEBMI had a 27% higher risk of death than those covered by UEBMI (HR: 1.27; 95% CI: 1.12–1.46; *p* < 0.001; Table 2). Other independent factors associated with OS included ALBI II (HR: 1.46; 95% CI: 1.26–1.70; *p* < 0.001), advanced stage (HR: 1.45; 95% CI: 1.15–1.83; *p* = 0.002), Milan criteria (HR: 0.67; 95% CI: 0.48–0.96; *p* = 0.026), major hepatectomy (HR:1.23, 95% CI: 1.05–1.43; *p* = 0.009), capsular invasion (HR: 1.28; 95% CI: 1.11–1.48; *p* = 0.001), MVI (HR: 1.76; 95% CI: 1.53–2.04; *p* < 0.001), satellite nodules (HR: 1.49; 95% CI: 1.25–1.78; *p* < 0.001), and PVTT (HR: 1.55; 95% CI: 1.20–1.99; *p* < 0.001; Table 2).

**TABLE 2:** Cox proportional hazards regression to predict overall survival based on Lasso regression.

Variables	HR	(95% CI)	*p* value
Non‐UEBMI (vs. UEBMI)	1.27	1.12–1.46	<0.001
ALBI II	1.46	1.26–1.70	<0.001
Tumor size, >5 cm	1.31	0.96–1.79	0.084
Advanced stage^a^	1.45	1.15–1.83	0.002
Milan criteria	0.67	0.48–0.96	0.026
Major hepatectomy	1.23	1.05–1.43	0.009
Capsular invasion	1.28	1.11–1.48	0.001
MVI	1.76	1.53–2.04	<0.001
Satellite nodules	1.49	1.25–1.78	<0.001
PVTT	1.55	1.20–1.99	<0.001

^a^ Advanced stage was defined as BCLC B/C stage.

**FIGURE 4:**
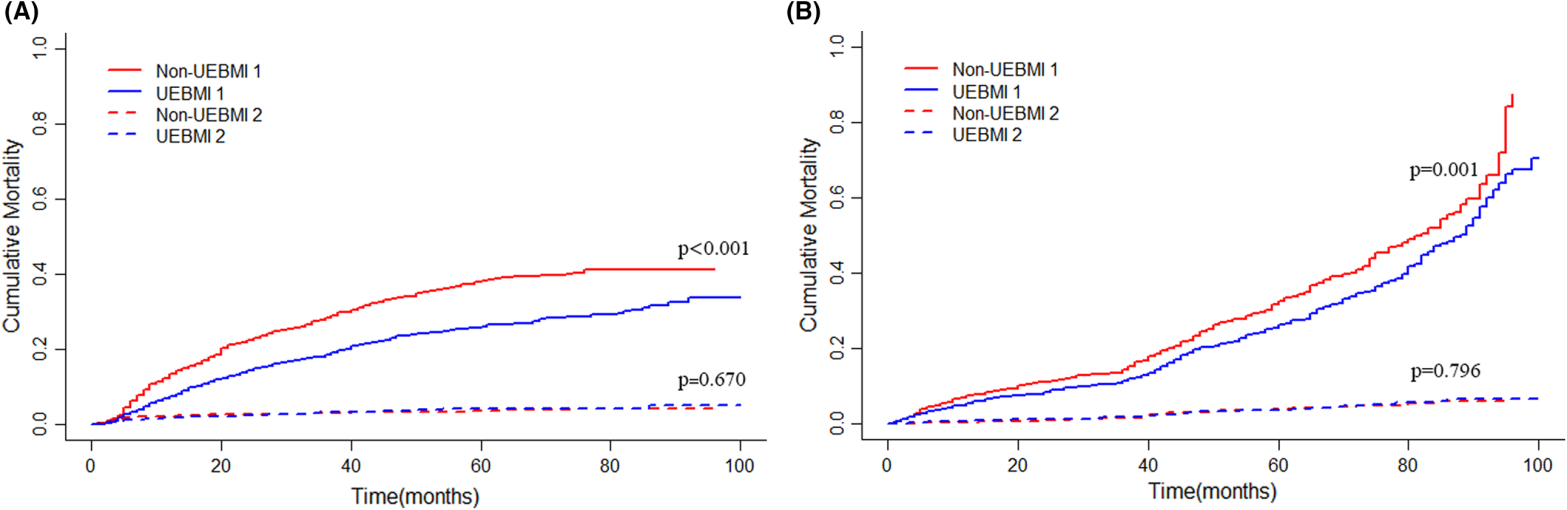
Competing risk analysis of CSS before (A) and after (B) PSM. 1, cumulative incidence of cancer‐specific death; 2, cumulative incidence of non‐cancer‐specific death.

During follow‐up, 894 patients experienced death, 792 from HCC and 102 from other causes. After controlling competitive events (non‐cancer‐specific death, before PSM: *p* = 0.670; after PSM: *p* = 0.796), patients in the UEBMI group had a lower cumulative incidence of CSS than those in the non‐UEBMI group before (Figure 4A, *p* < 0.001) and after PSM (Figure 4B, *p =* 0.001). Using a multivariable competing risk analysis based on the Fine and Gray model, independent factors associated with CSS were identified. In our model, the public insurance status of HCC patients after liver resection remained independently associated with CSS (non‐UEBMI vs. UEBMI; HR:1.36; 95% CI: 1.18–1.58; *p* < 0.001; Table 3). Other factors independently associated with CSS included ALBI II (HR: 1.20; 95% CI: 1.01–1.44; *p* = 0.043), smoke (HR:1.22; 95% CI: 1.02–1.44; *p* = 0.026), single tumor (HR: 0.70; 95% CI: 0.51–0.96; *p* = 0.024), Milan criteria (HR: 0.65; 95% CI: 0.45–0.92; *p* = 0.015), low differentiation (HR: 1.26; 95% CI: 1.03–1.55, *p* = 0.025), capsular invasion (HR: 1.31; 95% CI: 1.12–1.54; *p* = 0.001), MVI (HR: 1.69; 95%CI: 1.44–1.99; *p* < 0.001), satellite nodules (HR: 1.48; 95% CI: 1.21–1.81; *p* < 0.001), and PVTT (HR: 1.92; 95% CI: 1.34–2.76; *p* < 0.001).

**TABLE 3:** Multivariable Fine and Gray proportional hazard models of cancer‐specific survival.

Variables	HR	(95% CI)	*p* value
Non‐UEBMI (vs. UEBMI)	1.36	1.18–1.58	<0.001
ALBI II	1.20	1.01–1.44	0.043
Smoke	1.22	1.02–1.44	0.026
Single tumor	0.70	0.51–0.96	0.024
Milan criteria	0.65	0.45–0.92	0.015
Low differentiation	1.26	1.03–1.55	0.025
Capsular invasion	1.31	1.12–1.54	0.001
MVI	1.69	1.44–1.99	<0.001
Satellite nodules	1.48	1.21–1.81	<0.001
PVTT	1.92	1.34–2.76	<0.001

## 4 DISCUSSION

We demonstrated that underinsured HCC patients (non‐UEBMI) after liver resection had worse survival outcomes (OS/CSS) in this population‐based study in China. The public health insurance status of HCC patients after liver resection continued to be independently linked with OS and CSS even after controlling for clinicopathological variables and demographic variables. Disparities in the treatment received by recurrent HCC patients covered by different public insurance may to some extent explain the differences in survival outcomes.

Similar results have been reported in other cancers. Fang et al. demonstrated that locally advanced gastric cancer patients undergoing gastrectomy with limited or no insurance had a worse OS.^13^ Deol et al. found that robotic‐assisted lobectomy patients with private insurance had a better prognosis than those with combined and public insurance.^15^ In the Korean research on esophageal cancer, patients in the Medicare group had a lower 5‐year survival compared to those in the health insurance group.^14^ However, most of these studies have focused on the prognostic impact between different types of health insurance (e.g., private health insurance, public health insurance, Medicaid, and no insurance), whereas few studies have examined the internal differences in prognosis within the same type of health insurance, and the health insurance system may vary from country to country, which means that the conclusions of similar studies may vary across countries.^20^ As the largest developing country, the public health insurance system is the main medical insurance system in China, which greatly reduces the economic pressure on patients.^12^ Our research revealed that the public health insurance status of HCC patients after liver resection might be a factor that cannot be overlooked for improving their prognosis. Continued reform of health care systems in developing nations may reduce prognosis disparities among HCC patients after liver resection.

Several mechanisms may explain the observed survival difference between the two groups. First, health insurance status may affect patients' access to medical services.^21^ Studies have shown that underinsured patients were less likely to be screened and monitored for cancer and therefore were more likely to be diagnosed with advanced tumors, which might be associated with a poorer survival prognosis.^22^, ^23^, ^24^ HCC patients after liver resection have a very high recurrence rate, making regular follow‐up and monitoring of postoperative HCC patients very important.^25^ Regular postoperative monitoring and follow‐up can improve the chances of recurrent HCC patients receiving potentially curative therapy, which results in better survival outcomes.^26^, ^27^ In our study, more recurrent HCC patients in the UEBMI group received curative therapy (Table 4), which implied that they had a lower tumor burden at the time of recurrence. Underinsured HCC patients who undergo hepatectomy may lack regular postoperative monitoring and follow‐up, which may lead to a diagnosis of advanced tumors at the time of recurrence and worse survival outcomes.^21^, ^28^, ^29^ Second, health insurance status may influence the treatment choice of patients.^29^, ^30^, ^31^ Fighting cancer is not only a psychological and physical burden for cancer patients but also an inevitable financial burden.^4^, ^32^ For HCC patients who have already undergone primary hepatectomy, postoperative follow‐up monitoring, treatment for underlying liver disease, treatment for preventing recurrence, or treatment for recurrence remains a significant financial burden.^33^ There are significant differences between the benefits of various public health insurance programs due to differences in funding and management.^18^, ^19^ This means that patients with the same disease burden face varying financial burdens.^34^ It is possible for underinsured recurrent HCC patients to refuse or change from effective treatment on their own for financial reasons, which may impact their survival outcomes.^35^ In our study, recurrent HCC patients in the non‐UEBMI group were more likely to choose supportive care (Table 4). Moreover, postoperative adjuvant therapy is very important in cancer patients to prevent recurrence, however, adjuvant therapy in postoperative hepatocellular carcinoma is still controversial. The failure of the STORM study suggested that the role of targeted agents needs to be further explored in the field of postoperative adjuvant therapy for HCC patients. A randomized controlled study demonstrated that postoperative adjuvant TACE reduced tumor recurrence and improved recurrence‐free survival for HBV‐related HCC patients with a high risk of recurrence,^36^ and this treatment strategy was included in the guidelines for the diagnosis and treatment of hepatocellular carcinoma in China.^2^ In our study, HCC patients in the non‐UEBMI group had higher tumor burden but were less likely to receive adjuvant TACE (Table 1), which implied that some underinsured patients chose to refuse postoperative adjuvant TACE because of finical burden, which resulted in poor survival outcomes. Surgical intervention is an important means of achieving long‐term survival for patients with either primary or recurrent HCC.^37^ Sobotka et al. found that HCC patients with government‐funded insurance were less likely to undergo curative procedures such as liver transplantation and surgical resection.^31^ Zaydfudim et al. also reported that health insurance status might influence the therapy option for HCC patients even after adjusting for tumor stage.^29^ For recurrent HCC patients not amenable to surgical intervention, although advances in nonsurgical treatment (e.g., systemic therapy) can improve their prognosis to some extent,^38^ this can also impose a significant financial burden on them. Underinsured patients may not be able to afford the economic toxicity of cancer treatment.^32^ In our study, a lower proportion of recurrent HCC patients in the non‐UEBMI group received systemic therapy than those in the UEBMI group (Table 4). Previous studies have also shown that underinsured or no insurance patients were less likely to receive targeted therapy and immunotherapy.^39^, ^40^, ^41^

**TABLE 4:** Treatment modalities for patients with recurrent hepatocellular carcinoma.

Treatment of recurrence	UEBMI (*N* = 754)	Non‐UEBMI (*n* = 734)	*p* value
Curative therapy^a^	321 (42.6%)	244 (33.2%)	<0.001
Repeat liver resection	185 (24.5%)	154 (21.0%)	
Radiofrequency ablation	115 (15.3%)	85 (11.6%)	
Liver transplantation	21 (2.8%)	5 (0.7%)	
TACE	254 (33.7%)	272 (37.1%)	0.174
Systemic therapy	114 (15.1%)	83 (11.3%)	0.030
Other treatment	12 (1.6%)	19 (2.6%)	0.178
Supportive care^b^	5 3(7.0%)	116 (15.8%)	<0.001

^a^ Curative therapy includes repeat liver resection, radiofrequency ablation, and liver transplantation.

^b^ Patients who were unable or refused to receive the aforementioned treatments were managed by supportive care.

This is the first study to investigate the relationship between public health insurance status and the prognosis of HCC patients after liver resection in a developing country. Our study suggested that underinsured HCC patients after liver resection had worse survival outcomes, and less access to care for underinsured patients may explain the difference in survival. We also admit that the relationship between public health insurance status and the prognosis of HCC patients after liver resection is complex and that the underlying mechanisms require additional investigation. In summary, our conclusions provide a novel strategy for improving disparities in survival outcomes of HCC patients after liver resection and may also provide some basis for the reform of the health care system.

There are several limitations in this study. First, there is an unavoidable selection bias due to the retrospective nature of our study. HCC patients in our center are mainly from Sichuan Province, and in most regions of China, patients enrolled in UEBMI always enjoy better benefit packages. Consequently, our findings may, to some extent, represent variations in the survival of HCC patients who received hepatectomy covered by public health insurance in China. Second, because of the lack of relevant data, other socioeconomic factors (e.g., education level, income, occupation, residence) and the presence of combined supplementary insurance (e.g., commercial insurance, private insurance) were not included in this study. These factors may differ between the UEBMI and non‐UEBMI groups, which may reduce the influence of public health insurance status on the OS/CSS of HCC patients after liver resection. Future research should fully incorporate these variables to better comprehend how public health insurance status influences the OS/CSS of HCC patients following hepatectomy. Further research using richer data is required to fully comprehend the differences in survival outcomes of HCC patients after liver resection explored in our research.

## 5 CONCLUSIONS

This study demonstrated that underinsured HCC patients (non‐UEBMI) after liver resection had worse survival outcomes. Less access to care for underinsured patients may explain the difference in survival, but the corresponding conclusions need to be further explored.

## AUTHOR CONTRIBUTIONS

**Zhancheng Qiu:** Data curation (equal); methodology (lead); software (equal); writing – original draft (lead); writing – review and editing (equal). **Weili Qi:** Data curation (equal); methodology (equal); writing – review and editing (equal). **Youwei Wu:** Data curation (equal); resources (equal). **Lingling Li:** Methodology (supporting); resources (equal). **Chuan Li:** Conceptualization (lead); methodology (equal); writing – review and editing (lead).

## FUNDING INFORMATION

Funding for this study was provided by the National Natural Science Foundation of China youth fund (81900576). The funders had no role in the study design, data collection and analysis, decision to publish, or analysis of the results.

## ETHICS STATEMENT

The West China Hospital's Ethics Committee gave its approval to this study. The committee decided not to require informed consent because the study was retrospective (IRB No. 2022‐1468).

## Data Availability

The corresponding author will, upon reasonable request, share the datasets used and/or analyzed in the present research.
